# Supplementary material for: Induction of Hepatitis E Virus Anti-ORF3 Antibodies from Systemic Administration of a Muscle-Specific Adeno-Associated Virus (AAV) Vector
Source: Viruses. 2022 Jan 27;14(2):266. doi: 10.3390/v14020266 (PMC8878420; doi:10.3390/v14020266)
Supplement: Supplementary file 1 [file viruses-14-00266-s001.zip › viruses-1560766-supplementary.pdf]

viruses

MDPI

## Supplementary information for:

# Induction of hepatitis E virus anti-ORF3 antibodies from systemic administration of a muscle-specific Adeno-associated virus (AAV) vector

**Lars Maurer, Jihad El Andari, Kleopatra Rapti, Laura Spreyer, Eike Steinmann, Dirk Grimm and Viet Loan Dao Thi**

| **ORF3** | |
|---|---|
| ORF3 fd | 5′-GGTGGTTTCTGGGGTGAC-3′ |
| ORF3 rev | 5′-AGGGGTTGGTTGGATGAA-3′ |
| ORF3 Probe | FAM-TGATTCTCAGCCCTTCGC-BHQ1 |
| **POLR2A** | |
| Primer/probe mix | Mm00839502_m1 (Thermo Fisher Scientific, Waltham, MA, USA) |
| **eCMV (CMV enhancer) for AAV titration** | |
| CMVenh_fd | AACGCCAATAGGGACTTTCC |
| CMVenh_rev | GGGCGTACTTGGCATATGAT |
| CMVenh_Probe | FAM-CGGTAAACTGCCCACTTGGCAGT-BHQ1 |

**Supplemental Table S1.** Primers and probes for HEV ORF3 [1] and AAV genome quantification [2]. POLR2A, RNA polymerase II subunit A.

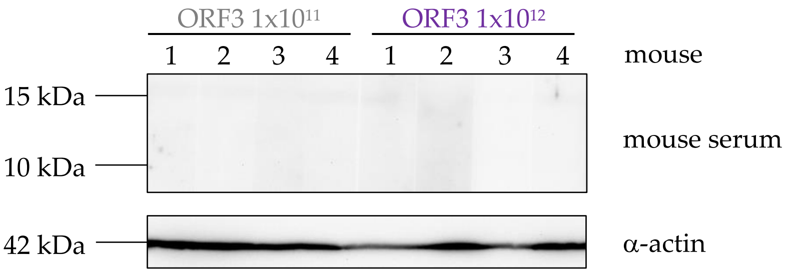


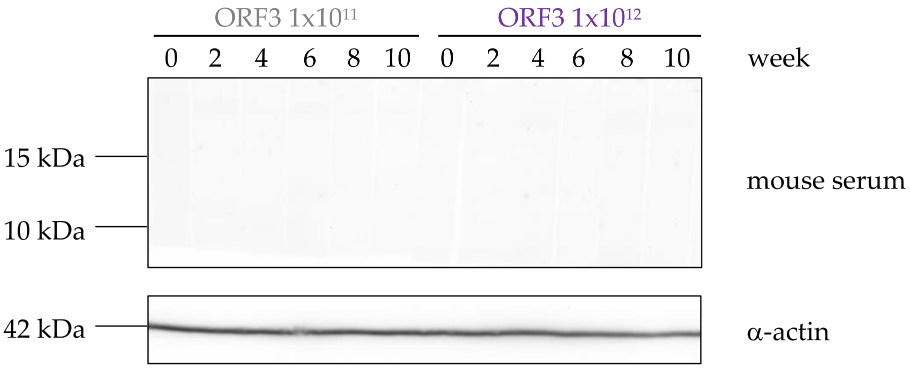


**Supplemental Figure S1.** Mock-transfected HEK-293 control membranes were incubated with sera from mice transduced with the indicated amounts of ORF3 AAV particles (upper blot corresponds to the control of Fig. 3A, lower blot to the control of Fig. 3B).

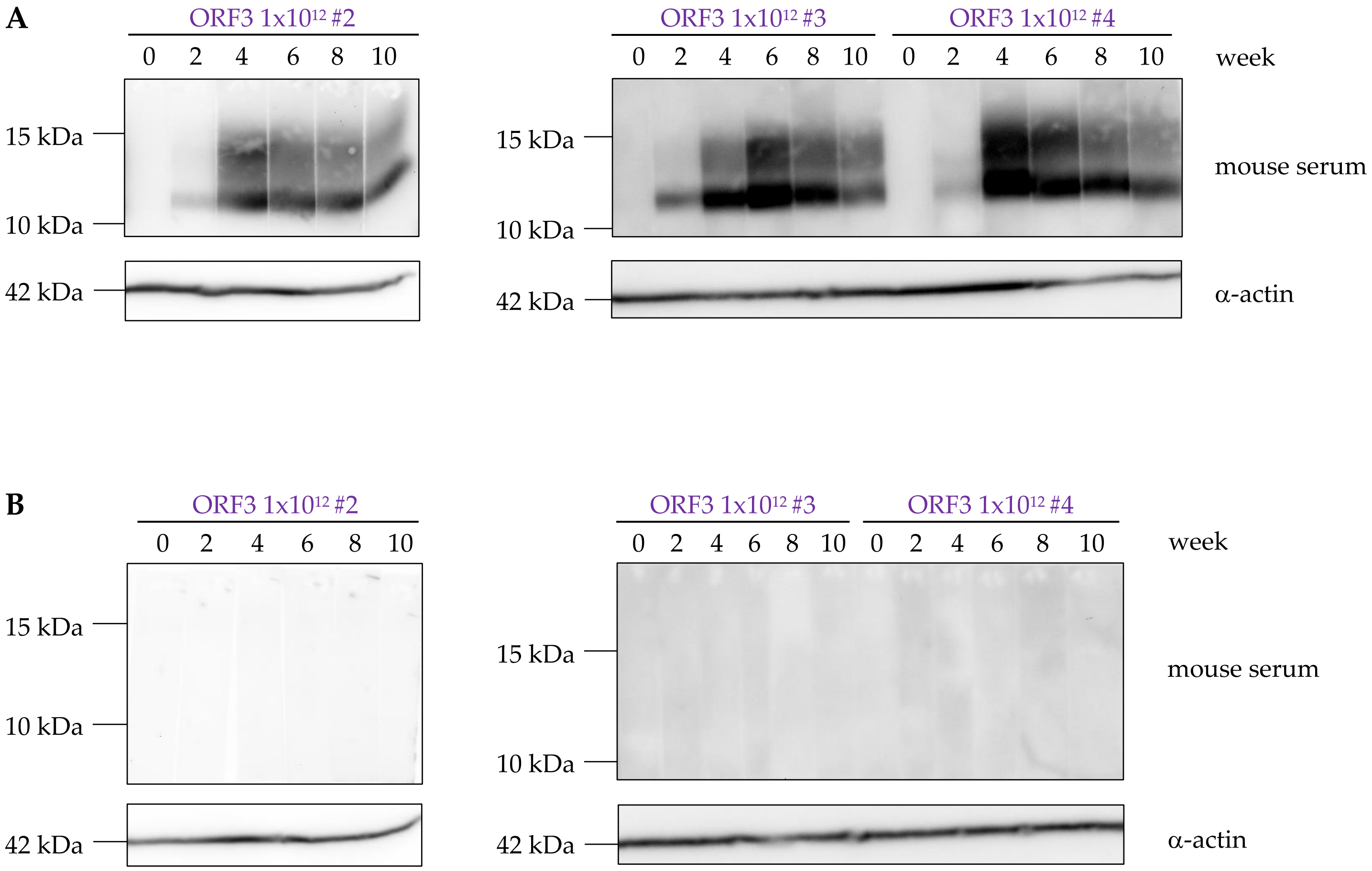


**Supplemental Figure S2.** Dynamics of antibody induction in remaining mice (1x10$^{12}$ ORF3 AAV) over 10 weeks. (**A**) HEK-293 cells transfected to express ORF3 were lysed and loaded onto a 12% SDS-PAGE. After separation and transfer, the membrane was cut into individual pieces and incubated with sera from mouse #2, #3, #4 (1x10$^{12}$ ORF3 AAV) harvested at the indicated different time points post-AAV injection, followed by a secondary anti-mouse antibody. As a loading control, actin was detected using an anti-actin mAb. (**B**) Mock-transfected HEK-293 control membranes were incubated with sera from the indicated mice (corresponding controls to the blots directly above). ORF3: = ~11 kDa, palmitoylated ORF3 = ~15 kDa, actin = ~42 kDa.

1. Jothikumar, N.; Cromeans, T.L.; Robertson, B.H.; Meng, X.J.; Hill, V.R. A broadly reactive one-step real-time RT-PCR assay for rapid and sensitive detection of hepatitis E virus. *J. Virol. Methods* **2006**, *131*, 65-71.
2. Fakhiri, J.; Schneider, M.A.; Puschhof, J.; Stanifer, M.; Schildgen, V.; Holderbach, S.; Voss, Y.; El Andari, J.; Schildgen, O.; Boulant, S.; et al. Novel Chimeric Gene Therapy Vectors Based on Adeno-Associated Virus and Four Different Mammalian Bocaviruses. *Mol. Ther. Methods Clin. Dev.* **2019**, *12*, 202-222.
